# Fully covered self-expandable metal stents (SEMS), partially covered SEMS and self-expandable plastic stents for the treatment of benign esophageal ruptures and anastomotic leaks

**DOI:** 10.1186/1471-230X-12-19

**Published:** 2012-02-29

**Authors:** Petra GA van Boeckel, Kulwinder S Dua, Bas LAM Weusten, Ruben JH Schmits, Naveen Surapaneni, Robin Timmer, Frank P Vleggaar, Peter D Siersema

**Affiliations:** 1 Department of Gastroenterology & Hepatology, University Medical Center Utrecht, Room F02.618, Heidelberglaan 100, 3584 CX Utrecht, The Netherlands; 2 Department of Gastroenterology and Hepatology, Medical College of Wisconsin, Milwaukee, WI, USA; 3 Department of Gastroenterology and Hepatology, Antonius Ziekenhuis Nieuwegein, Nieuwegein, The Netherlands

## Abstract

**Background:**

Benign esophageal ruptures and anastomotic leaks are life-threatening conditions that are often treated surgically. Recently, placement of partially and fully covered metal or plastic stents has emerged as a minimally invasive treatment option. We aimed to determine the clinical effectiveness of covered stent placement for the treatment of esophageal ruptures and anastomotic leaks with special emphasis on different stent designs.

**Methods:**

Consecutive patients who underwent placement of a fully covered self-expandable metal stent (FSEMS), a partially covered SEMS (PSEMS) or a self-expanding plastic stent (SEPS) for a benign esophageal rupture or anastomotic leak after upper gastrointestinal surgery in the period 2007-2010 were included. Data on patient demographics, type of lesion, stent placement and removal, clinical success and complications were collected

**Results:**

A total of 52 patients received 83 esophageal stents (61 PSEMS, 15 FSEMS, 7 SEPS) for an anastomotic leak (n = 32), iatrogenic rupture (n = 13), Boerhaave's syndrome (n = 4) or other cause (n = 3). Endoscopic stent removal was successful in all but eight patients treated with a PSEMS due to tissue ingrowth. Clinical success was achieved in 34 (76%, intention-to-treat: 65%) patients (PSEMS: 73%, FSEMS: 83%, SEPS: 83%) after a median of 1 (range 1-5) stent and a median stenting time of 39 (range 7-120) days. In total, 33 complications in 24 (46%) patients occurred (tissue in- or overgrowth (n = 8), stent migration (n = 10), ruptured stent cover (all Ultraflex; n = 6), food obstruction (n = 3), severe pain (n = 2), esophageal rupture (n = 2), hemorrhage (n = 2)). One (2%) patient died of a stent-related cause.

**Conclusions:**

Covered stents placed for a period of 5-6 weeks may well be an alternative to surgery for treating benign esophageal ruptures or anastomotic leaks. As efficacy between PSEMS, FSEMS and SEPS is not different, stent choice should depend on expected risks of stent migration (SEPS and FSEMS) and tissue in- or overgrowth (PSEMS).

## Background

Esophageal ruptures and anastomotic leaks are life-threatening injuries with a high mortality rate [1-7]. Most ruptures occur after (endoscopic) instrumentation or surgery, i.e. leaking anastomosis, but may also occur spontaneously after vomiting (Boerhaave's syndrome) [8]. Surgical repair including surgical closure or cervical exclusion or esophagectomy has long been considered to be 'gold standard' [4,8-12]. Although surgery improves survival, it has been reported to be associated with a high mortality rate (30%), especially after a delayed diagnosis [2].

Endoscopic stent placement is a well accepted and effective treatment for malignant dysphagia [13]. Recently, temporary endoscopic stent placement, either with fully (FSEMS) or partially (PSEMS) covered self-expanding metal stents or a self-expanding plastic stent (SEPS), has emerged as a minimally invasive treatment option for benign esophageal ruptures and leaks. A favorable outcome with low morbidity and mortality has been reported [14-19]. Stents are able to effectively seal leaks and offer protection of the esophageal mucosal wall while healing takes place when adequate drainage of fluid collections from the mediastinum or pleural cavity is concurrently performed. The main drawbacks of stent placement are tissue in- or overgrowth and stent migration necessitating reinterventions. Particularly, reactive nonmalignant tissue in- or overgrowth and embedding of the stent in the esophageal wall may be a problem, especially when partially covered stents are left in place for a longer duration. Endoscopic stent removal in case of severe stent embedding may cause esophageal perforation [20]. On the other hand, migration rates are higher when fully covered stents, either SEMS or SEPS, are used [21-23].

Experience with temporary stenting for nonmalignant esophageal ruptures or anastomotic leaks is limited and studies comparing surgery with stent placement have not been performed. In addition, studies comparing FSEMS, PSEMS and SEPS for the treatment of benign esophageal ruptures and leaks are not available.

In this study, we evaluated safety and clinical effectiveness of treating benign esophageal ruptures and anastomotic leaks with covered stents, with special emphasis on different stent designs.

## Methods

### Patients

All patients who had received a self-expandable metal or plastic stent for sealing a benign esophageal rupture or anastomotic leak after esophageal or gastric surgery in the period January 1, 2007-January 1, 2010 were enrolled in this study. Data on patient demographics, type and cause of lesion, stent type, placement and removal details, clinical success (sealing rate), complications, reinterventions and mortality were retrospectively collected. Patients with malignant fistulas or ruptures, or for whom no follow-up information was available were excluded (10%).

### Esophageal stents

All patients received a covered esophageal stent, a PSEMS, FSEMS or SEPS (see below).

The PSEMS used in our study were the:

- Ultraflex stent (Boston Scientific, Natick, MA), length 120 mm, cover 90 mm, diameter 28/23 mm, or length 150 mm, cover 120 mm, diameter 23/18 mm;

- WallFlex Esophageal Stent (Boston Scientific), length 120 mm, cover 90 mm, diameter 28/23 mm.

The FSEMS used were the:

- SX-ELLA Stent Esophageal HV (ELLA-CS, Hradec Králové, Czech Republic), length 85 mm, diameter 25/20/25 mm or length 110 mm, diameter 25/20/25 mm;

- ALLIMAX-E Esophageal stent (Alveolus, Charlotte, NC), length 120 mm, diameter 22 mm;

- Choo stent (M.I. Tech, Seoul, South Korea), length 60 mm, diameter 18 mm.

The SEPS used was the Polyflex Esophageal Stent (Boston Scientific), length 90 mm, diameter 25/21 mm.

Endoscopic stent placement was performed under fluoroscopic control. Endoscopic stent removal was performed with a rat-tooth forceps grasping the proximal end of the stent; only some of the Ultraflex stents were grasped distally, resulting in removal of an inverted stent. When endoscopic stent removal was expected to be complicated due to tissue ingrowth (PSEMS) and/or overgrowth (all stent types), a FSEMS of the same size was placed inside the stent. This stent at least overlapped the previously placed stent and induced pressure necrosis of the tissue in- or overgrowth. This resulted in uncomplicated removal of both stents after 10-14 days (stent-in-stent method) [24]. After stent removal, an endoscopy and/or a water-soluble contrast esophagogram was performed to confirm sealing. All endoscopic procedures were performed under conscious sedation (midazolam or propofol) or general anesthesia according to the patient's condition.

### Endpoints

Primary endpoint of the study was clinical success defined as sealing of a rupture or leak as confirmed by endoscopy and an additional esophagogram in case of doubt. Secondary outcomes included technical success of stent placement and removal, complication rates and survival. For technical outcome we registered details on stent deployment and placement at the required location. Removal was considered to be successful when the stent could be removed as a whole and without complications in one session. Complications included stent- and procedure-related adverse events.

### Statistical analysis

The following variables were included in the analyses: a) clinical characteristics: age, gender, lesion length, location and etiology, and prior treatment, b) outcome and survival: technical success, clinical success, survival and cause of death, and c) complications. Results were expressed as mean ± SD and medians with range, as appropriate. Chi-Square test and Kruskal Wallis test were used as appropriate. All analyses were performed on an intention-to-treat (ITT) basis. A p-value <0.05 was considered statistically significant. Statistical analyses were conducted using SPSS version 15 (SPSS Inc, Chicago, Ill. USA).

## Results

### Clinical characteristics

In total, 52 patients treated with 83 covered self-expandable stents were included in three different hospitals (University Medical Center Utrecht n = 25, Medical College of Wisconsin, Milwaukee n = 15 and Antonius Ziekenhuis Nieuwegein n = 12). Clinical characteristics of the patients are shown in Table 1. More than half of the patients had an anastomotic leak after gastrectomy with esophagojejunostomy (n = 15), (transhiatal) esophagectomy with gastric tube formation (n = 9), gastric bypass (n = 6) or resection of an esophageal diverticulum (n = 1). Iatrogenic esophageal ruptures occurred during the following procedures: pneumatic dilation (n = 6), tracheal intubation (n = 3), esophageal stenting for benign strictures (n = 2), Nissen fundoplication (n = 1), a Belsey Mark IV procedure (n = 1) and medianoscopic biopsy taking (n = 1). Other causes included Boerhaave syndrome (n = 4), a rupture following radiation therapy (n = 1), spontaneous rupture above an impacted food bolus (n = 1) and disruption of a mediastinal abscess (n = 1). Most patients (n = 41 (79%)) received antibiotic treatment. In 24 (46%) patients, concurrent drainage of the pleural cavity (n = 12), mediastinum (n = 4) or both (n = 8) was performed either surgically (n = 18 (75%)) or radiologically (n = 6 (25%)).

**Table 1:** Clinical characteristics of 52 patients treated with an esophageal stent for a benign rupture or anastomotic leak

Characteristic	n = 52
Age, year (mean ± SD)	60 (±14)

Gender, number of patients (%)	

Male	32 (61)
Female	20 [39]

Cause of benign rupture or leak, number of patients (%)	

Anastomotic	32 (62)
Iatrogenic	13 [25]
Boerhaave's syndrome	4 [8]
Other	3 [5]

Location of benign rupture or leak, number of patients (%)	

Distal esophagus	13 [25]
Mid-esophagus	24 (45)
Proximal esophagus	11 [21]
Unknown	4 [9]

Length of rupture or leak, cm (median (range))	2 (0.2-7)

Time interval between rupture and stent placement, number of patients (%)	

Within 24 hours	5 [10]
After 24 hours	47 (90)

Prior treatment for benign rupture or leak, number of patients (%)	

Stent placement in another hospital	3 [5]
Surgery	3 [5]
Clip placement	1 [2]

None	45 (88)

Antibiotic treatment, number of patients (%)	

Yes	41.(79)
No	11 [21]

Concurrent fluid drainage, number of patients (%)	
Yes	24 (46)
No	28 (54)

Total days of treatment with a stent, median (range)	39 (1-742)

In total, 83 stents (median 1, range 1-10) were placed, of which 61 (74%) were PSEMS, 15 (18%) FSEMS and 7 (8%) SEPS. The median number of days of stent placement was 39 days (range 1-742). In one patient with an anastomatic leak after (transhiatal) esophagectomy, a total of 10 stents was placed resulting in a total stenting time of 742 days.

Median follow up was 470 days (range 25-1200 days).

### Stent placement and removal

Eighty-two of 83 (99%) stents were successfully placed (Table 2). In one patient, a PSEMS was placed too proximally and could not be repositioned. Therefore, a second PSEMS was placed inside the stent during the same procedure which successfully covered the leak.

**Table 2:** Outcome and survival of 52 patients treated with 83 esophageal stents for a benign perforation or anastomotic leak

Characteristic	
Technically successful stent placement, number of stents (%)	82/83 (99)

Technically successful stent removal, number of stents (%)	63/71 (89)

Reasons for stent removal, number of stents (%)	
Scheduled	52 (73)
Early migration	9 [13]
Leakage through ruptured stent cover	6 [8]
Tissue in- and/or overgrowth	2 [3]
Severe pain	2 [3]

Clinical success, number of patients (%)	34 (65)

Cause of death, number of patients (%)	
Rupture or leakage	4 [8]
Stent	1 [2]
Not related to rupture/leakage or stent placement	2 [4]

In total, 71 (86%) stents were endoscopically removed after a median of 25 (range 1-197) days (PSEMS 24 (1-197) days, FSEMS 23 (1-120) days, SEPS 42 (14-90) days) (*p *= 0.50). Of these, 52 were removed according to the scheduled treatment plan, while the other 19 were removed earlier due to the occurrence of complications (Table 2). Endoscopic stent removal was successful in all but eight patients with a PSEMS due to tissue in- and/or overgrowth. In four of these patients, a FSEMS was placed inside the PSEMS to achieve pressure necrosis, after which the stent could be removed successfully (stent-in-stent method) [24]. In one patient, the stent was successfully removed during a follow-up endoscopic procedure 4 days later. In one patient esophagectomy was performed for removal of the stent. In two patients, a rupture occurred during stent removal, which necessitated placement of another stent during the same procedure to seal the rupture. These two stents could be removed uneventfully, 17 and 23 days after placement.

### Outcome and survival

**Figure 1:**
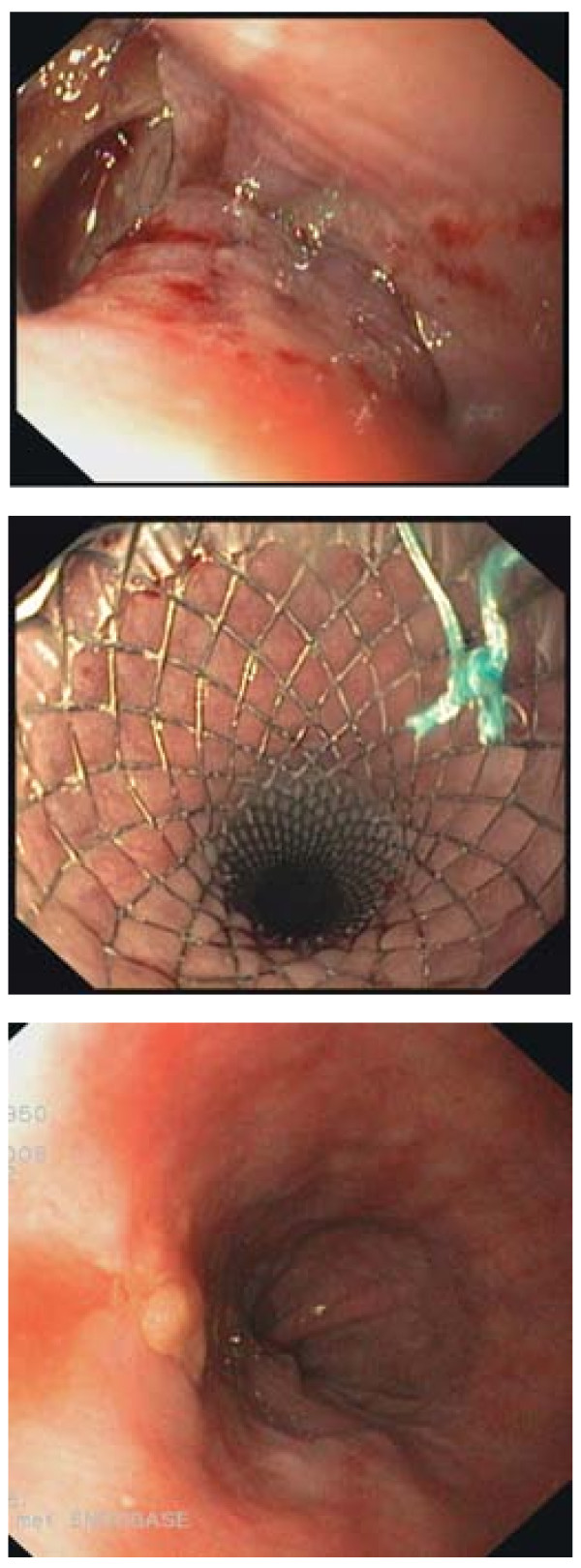
**Endoscopic view: A Iatrogenic rupture following pneumodilation, B Partially covered metal stent placed in the esophageal lumen sealing the rupture, C Healed rupture after stent removal**.

Clinical success was achieved in 34 (76%, ITT: 65%) patients (PSEMS: 73%, ITT: 69%; FSEMS: 83%, ITT: 56%; SEPS: 83%, ITT: 71%, *p *= 0.33) after a median of 1 (range 1-5) stent and a median stenting time of 39 (range 7-120) days (Figure 1, Table 2). Of the other 18 patients, 4 patients underwent surgical treatment (3 esophagectomy, 1 surgical repair), 2 patients had further conservative treatment, 7 died before stent removal. One (2%) patient treated with FSEMS died from a stent-related death (severe hemorrhage); this patient refused further interventions. Another four patients died from rupture-related causes (sepsis), one patient from underlying malignant disease and one patient from active euthanasia.

### Complications

In total, 33 complications in 24 patients (46%) occurred (tissue in- and/or overgrowth (n = 8), stent migration (n = 10), ruptured stent cover (n = 6), food obstruction (n = 3), severe retrosternal pain (n = 2), esophageal rupture due to stent removal (n = 2) and hemorrhage (n = 2)) (Table 3). Stent migration occurred most frequently with FSEMS (20%), followed by SEPS (14%) and PSEMS (10%), while tissue in- and/or overgrowth was only seen with PSEMS (11%). Ruptured stent covers were only seen with Ultraflex stents. In addition, severe pain and food obstruction were also only seen in patients treated with PSEMS. In both patients with unbearable pain, the stent was removed after 1 and 6 days. One patient underwent surgical treatment (n = 1), while the other patient had conservative treatment (n = 1). One hemorrhage occurred with a FSEMS and the other with a PSEMS. One patient died (see above), while the hemorrhage in the other patient was treated successfully with adrenaline injections. Two esophageal ruptures occurred during removal of PSEMS. Ruptures occurred at the site of the uncovered stent meshes and these were treated with a second stent (see above).

**Table 3:** Complications in 52 patients treated with an esophageal stent for a benign rupture or anastomotic leak

Complication	Number (%)
Total complications	33 in 24 patients (46)

Stent migration	10

Tissue in- and/or overgrowth	8

Ruptured stent cover	6

Food obstruction	3

Hemorrhage	2

Severe pain	2

Ruptured esophagus (due to stent removal)	2

## Discussion

To our knowledge, this is the first study comparing different stent types, i.e. FSEMS, PSEMS and SEPS, for treatment of benign esophageal ruptures and leaks. Furthermore, our patients were included in a relatively short time compared to other cases series which makes a non-randomized comparison between different stent designs more reliable. Finally, all stents were placed in centers with a high level of expertise in stent placement.

Clinical success was achieved in 34 (76%) patients with no statistically significant differences between partially and fully covered metal and plastic stents (PSEMS: 73%, FSEMS: 83% and SEPS: 83%). This is in accordance with previous studies reporting successful sealing rates of 48%-100% with no obvious differences between PSEMS (48%-81%), FSEMS (48%-90%) and SEPS (67%-100%) [14-18,25-37]. The median stenting time to achieve healing in our study was 39 days, which was also not different from other studies, in which stenting time varied between 20 and 135 days [15,27,29,31,34,38]. Repeat endoscopy in asymptomatic patients with a stent in situ for assessment of healing was not routinely performed. Animal studies have suggested that a stenting time of 30 days should be enough for tissue healing [39]. Based on our results, we recommend that removal of esophageal stents placed for a rupture or leak should be performed after 5-6 weeks. However, further studies are however needed to determine the ideal sealing conditions, which include stenting time, type of stent to be used but also the extent of drainage of extra-esophageal fluid. In the presence of fluid collections in the pleural cavity or mediastinum, adequate resolution of these fluid collections is an absolute prerequisite for complete healing of an esophageal rupture or anastomotic leak. This can be done by endoscopic, radiologic or surgical means [34,40].

The complication rate of stent placement in our series, i.e., 33 complications in 24 patients (46%), was comparable to those reported in other series for this indication, i.e. 20%-72%. Major complications that have been reported include stent migration and tissue in- or overgrowth [14-17,27,28,33,36-38]. Stent migration occurred in 10 of 52 patients in our series. Stent migration was probably due to the fact that the far majority of these patients had no obstructive lesion which could aid in keeping the stent in place. Stent migration occurred most frequently with fully covered stents. This is due to the known reduced anchoring capacity of FSEMS and SEPS compared to PSEMS [23,41]. Fixating FSEMS of SEPS to the esophageal wall with an endoscopic clip has been shown to be effective in preventing migration [42]. In our study no attempts were made to endoscopically anchor the stent.

In contrast, tissue in- or overgrowth was exclusively seen with PSEMS (11%). Some endoscopists have a however a preference for PSEMS as it is thought that the normal esophageal tissue above and below the rupture or leak can project through the uncovered stent mesh, improving sealing quality and reducing the risk of stent migration. It has been shown that this hyperplastic tissue reaction results from a local fibrotic reaction and/or the proliferation of granulation tissue. This can already be seen as early as 14 days, but also at a later stage after stent placement [43]. In our patients, tissue in- or overgrowth occurred between 33 and 211 (median 40) days after stent placement. The type of stent material may play a causative role in the formation of hyperplastic tissue growth, with metal or nitinol stents being more prone to tissue growth than plastic stents [44]. Apart from this, it is probably also associated with the radial force and diameter of the stent, which may cause pressure on the esophageal wall and in that way induce a hyperplastic tissue reaction. Finally, duration of stenting is also a factor, with a prolonged stenting time increasing the risk. In our study, tissue in- and/or overgrowth complicated removal of PSEMS in 8 patients. When PSEMS are used for the treatment of benign ruptures or anastomotic leaks, we suggest to replace the stent on a 2-4 week basis. Alternatively, one may decide to leave the PSEMS longer in place, in which case a fully covered stent design of the same diameter can be placed inside the stent (stent-in-stent method), as was described in the Materials and Methods. This method allows uneventful removal of both stents after 10-14 days [24].

Our mortality rate (10%) was in the same range as that found in other studies (0-28%) [15-18,25-34,36-38]. Previous studies have reported that the time between onset of rupture or leak and performing an intervention is the most critical prognostic factor [2,10,12,18], with an increasing delay between rupture or leak and treatment being associated with a worse prognosis due to higher occurrence of (septic) complications. In our study, all patients that died had the stent placed more than 24 hours after the onset of rupture. Therefore, it seems clear that treatment, i.e., sealing the rupture or leak, should be performed as early as possible. The mortality rate (10%) associated with stent placement for this indication is likely to compare favorably with the mortality rate reported for surgical management (12%-50%) [18].

Until now, it is unclear which rupture or leak should be treated with stenting or primary surgery. Stenting has been proposed for ruptures or leaks that are smaller than 70% of the circumference [34], whereas surgery has been proposed for larger ruptures or leaks. The only true evidence would come from a randomized trial comparing these two treatment modalities, with special reference to the underlying disorder, extent and time since the rupture or leak occurred and severity of the extra-esophageal contamination. Nevertheless, the limited number of patients for such a trial and the promising results of stenting make such a trial difficult if not impossible to perform.

We are aware of the limitations of this study due to the retrospective design of the study. First, there was a variety of treatment protocols in our patients. In some patients, stent removal or exchange was performed at shorter intervals than in others and concurrent treatment, such as drainage of fluid collections, was also different between patients. Consequently, this could have affected clinical success rates, but also complication rates. Furthermore, selection bias cannot be excluded in our study, since there is still no guideline that clearly defines which patient benefits from stenting and which patient from surgery.

In conclusion, our results demonstrate that covered stents, placed for a period of 5-6 weeks, are a treatment option for sealing benign esophageal ruptures or leaks. As efficacy between PSEMS, FSEMS and SEPS was not different, stent choice should depend on expected risks of stent migration (SEPS and FSEMS) and tissue in- or overgrowth (PSEMS).

## Competing interests

P.G.A. van Boeckel, B.L.A.M. Weusten, R.J. H. Schmits, N. Surapaneni, R. Timmer and F.P. Vleggaar have no conflicts of interest or financial ties to disclose. S. Dua is consultant for Boston Scientific and Cook Medicals. P.D. Siersema is consultant, speaker and research recipient for Boston Scientific and speaker and research recipient for Cook Medicals.

## Authors' contributions

PB: study design, data collection, data analyses, data interpretation, writing of the manuscript. KD: data collection, data interpretation, manuscript editing. BW: data collection, manuscript editing, RS: data collection, data interpretation, manuscript editing, NS: data collection, manuscript editing, RT: manuscript editing, FV: data interpretation, manuscript editing, PS: study design, data interpretation, manuscript editing. All authors read and approved the final manuscript.

## Pre-publication history

The pre-publication history for this paper can be accessed here:

http://www.biomedcentral.com/1471-230X/12/19/prepub
